# Clinical Value of the Ottawa Ankle Rules for Diagnosis of Fractures in Acute Ankle Injuries

**DOI:** 10.1371/journal.pone.0063228

**Published:** 2013-04-30

**Authors:** Xin Wang, Shi-min Chang, Guang-rong Yu, Zhi-tao Rao

**Affiliations:** Department of Orthopedics, Tongji Hospital of Tongji University, School of Medicine, Shanghai, China; Universidad Europea de Madrid, Spain

## Abstract

**Background:**

The Ottawa ankle rules (OAR) are clinical decision guidelines used to identify whether patients with ankle injuries need to undergo radiography. The OAR have been proven that their application reduces unnecessary radiography. They have nearly perfect sensitivity for identifying clinically significant ankle fractures.

**Objectives:**

The purpose of this study was to assess the applicability of the OAR in China, to examine their accuracy for the diagnosis of fractures in patients with acute ankle sprains, and to assess their clinical utility for the detection of occult fractures.

**Methods:**

In this prospective study, patients with acute ankle injuries were enrolled during a 6-month period. The eligible patients were examined by emergency orthopedic specialists using the OAR, and then underwent ankle radiography. The results of examination using the OAR were compared with the radiographic results to assess the accuracy of the OAR for ankle fractures. Patients with OAR results highly suggestive of fracture, but no evidence of a fracture on radiographs, were advised to undergo 3-dimensional computed tomography (3D-CT).

**Results:**

183 patients with ankle injuries were enrolled in the study and 63 of these injuries involved fractures. The pooled sensitivity, specificity, positive predictive value and negative predictive value of the OAR for detection of fractures of the ankle were 96.8%, 45.8%, 48.4% and 96.5%, respectively. Our results suggest that clinical application of the OAR could decrease unnecessary radiographs by 31.1%. Of the 21 patients with positive OAR results and negative radiographic findings who underwent 3D-CT examination, five had occult fractures of the lateral malleolus.

**Conclusions:**

The OAR are applicable in the Chinese population, and have high sensitivity and modest specificity for the diagnosis of fractures associated with acute ankle injury. They may detect some occult fractures of the malleoli that are not visible on radiographs.

## Introduction

Foot and ankle injuries are common clinical conditions treated by emergency physicians; these injuries account for 6–12% of the patients seen in emergency departments (ED) [1]. Currently, almost all patients with foot and ankle injuries undergo radiographic examination to exclude fracture; however, fewer than 15% of these patients actually have fractures [2], thus most of these radiographs are unnecessary. Provision of these unnecessary radiographic examinations increases the demands on the health care system, and can result in prolonged patient waiting times. To decrease unnecessary radiation exposure and reduce the waiting time for patients in EDs, Stiell *et al.* developed clinical decision rules (termed the Ottawa ankle rules, OAR) for excluding fractures in acute ankle injuries using only physical examination [3]–[5]. Since their introduction in 1992, the OAR have been widely applied in many countries [6]–[13], and have been validated as highly sensitive and modestly specific for the detection of ankle fractures in multiple clinical settings [5]. However, there have been no studies of the clinical applicability of the OAR performed in the Chinese mainland population. This prospective study was designed to validate the applicability of the OAR in the Chinese population in Shanghai, and to evaluate the accuracy of the OAR for the diagnosis of fractures in acute ankle sprain patients in China.

In clinical practice, there are some fractures that cannot be detected on radiographs. Physical examination is very important for the detection of this type of fracture. 3D-CT has proven to be more sensitive for the diagnosis of these fractures than traditional radiographs. The other aim of this study was to assess the clinical utility of the OAR for the diagnosis of these occult fractures.

## Methods

### Study design

This prospective study was performed over a 6-month period, during which time all patients presenting with ankle injuries to the orthopedic ED of our teaching hospital at Tongji University in Shanghai were considered for enrollment. Upon admission to the emergency department for acute injury, the patient's history was taken and physical examination was performed. Findings were recorded on standardized examination forms to ensure consistency of data collection. The assessing physicians, all full-time orthopedic specialists in our ED, were trained in the definition and application of the OAR before the study. All of the physicians who participated in this study had more than 5 years of clinical experience in orthopedics.

**Figure 1:**
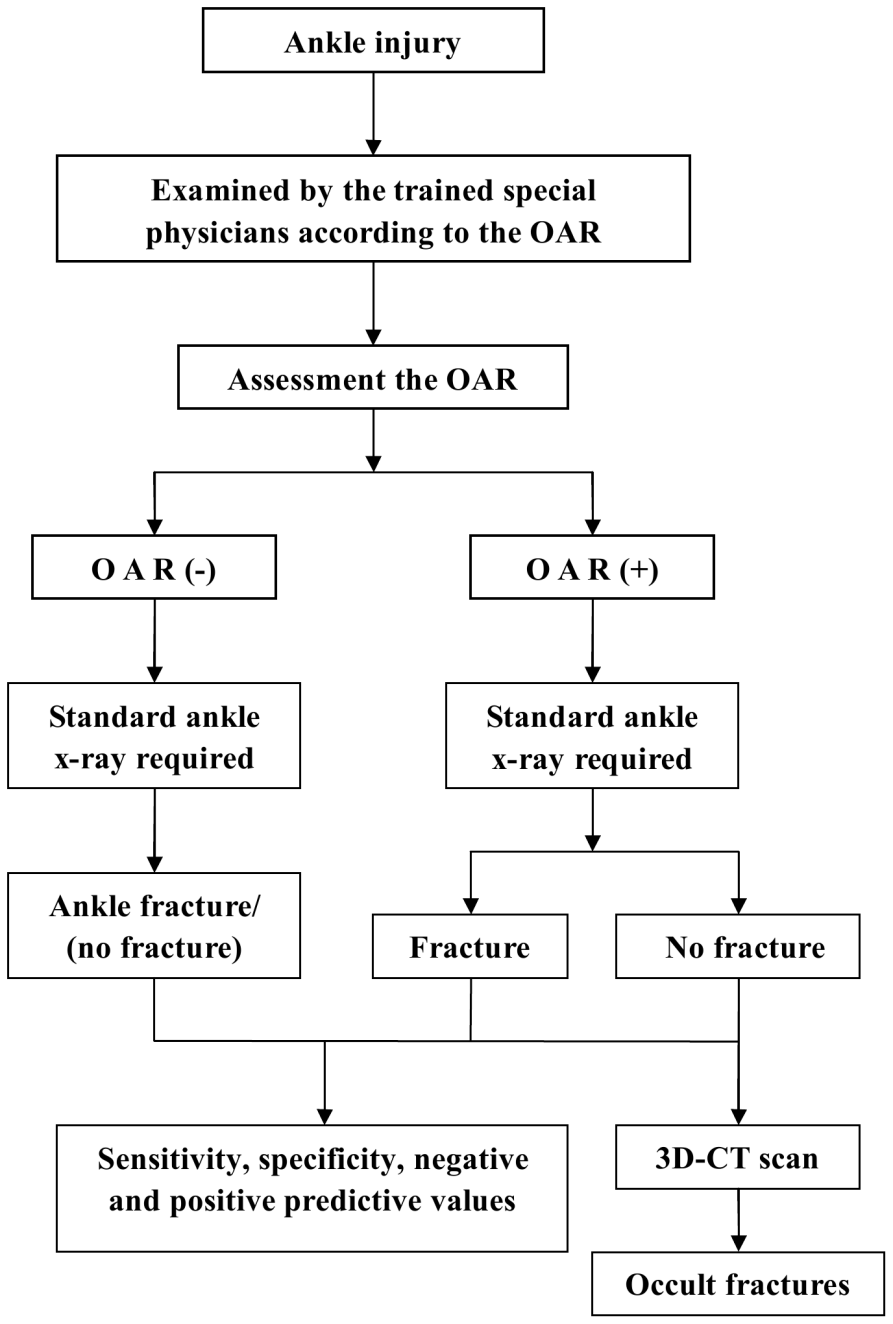
Schematic representation of the study protocol. The patients were examined by the specialist physicians trained in the application of the OAR. Subsequently, every observer filled in the standardized forms and assessed the OAR (+/−). All patients underwent radiography. Sensitivity and specificity of the OAR were calculated according to the radiography result. The patients with highly positive OAR, but negative radiography, were advised to undergo a 3D-CT scan.

After physical examination, every patient was asked to undergo standard radiographs of the injured ankle; these included antero-posterior and lateral views. All patient evaluations were completed before the results of the radiographs were obtained. The assessing physicians recorded their prediction of the likelihood of ankle fracture (positive or negative) for each patient based on the OAR examination results. The results of the radiographs were compared with the predictions based on the OAR. Patients with obvious soft-tissue swelling or a high fracture likelihood based on the OAR, but negative radiographic results, were advised to undergo a 3-dimensional computed tomography (3D-CT) scan to detect occult fractures (Figure 1). CT scans are particularly valuable for the diagnosis of occult fractures of the malleolus. All participants were given verbal information consent. Since 3D-CT is not part of the routine examination in cases of ankle sprain, the patients who underwent 3D-CT voluntarily agreed to the examination and were asked to pay for it. The study protocol was approved by the institutional review board of Tongji hospital.

### Study subjects

All patients with acute ankle injuries that presented to our hospital during the study period were enrolled if they met the inclusion criteria. The ankle area was defined as the malleolar area, which includes the distal 6 cm of the tibia and fibula and the entire malleolus to its distal tip. To be eligible for inclusion in this study, patients were required to be more than 18 years of age, to have presented to the ED within 72 h of injury to the ankle, to have a closed injury without a skin wound, and to be able to communicate without difficulty. Exclusion criteria included pregnancy, an open skin wound, more than 72 h having elapsed since the time of injury, obvious deformity of the ankle, and an altered sensorium or peripheral nerve disease. Ankle sprains as part of more severe trauma were also excluded from the study.

### Outcome parameters and statistical analysis

The radiographic results were compared with the likelihood of fracture predicted based on the OAR to calculate the sensitivity and specificity of the OAR for the diagnosis of fracture in acute ankle injuries in our patient population. The sensitivity and specificity of the OAR were calculated, as was the percentage reduction in the number of radiographs that would have been ordered had the OAR been followed. Negative and positive predictive values were calculated. The incidence rate of occult fractures detected by 3D-CT scan was calculated in patients with highly positive OAR results and negative radiographs.

## Results

A total of 197 patients with ankle injuries were initially enrolled during the study period. Fourteen patients were later excluded from the study; eight patients whose radiographs had been taken at another hospital before the OAR-based evaluation, and six whose clinical data forms were not completed by the assessing physicians. Ultimately, 183 eligible patients with acute ankle injuries were included in the study. These consisted of 81 male and 102 female patients ranging in age from 18 to 70 years (average age 36.6 years). Of these 183 patients with acute ankle injuries, 85 suffered injury while walking, 23 while running, 48 while going up or down stairs, and 27 while participating in sports.

Of the 183 ankle injuries seen, the emergency orthopedic specialists predicted based on the OAR that there were 126 patients with malleolar fractures. These patients, classified as positive by the OAR, are considered to require a series of standard ankle radiographs to confirm the diagnosis. Sixty-one fractures were radiographically confirmed in the 126 OAR-positive patients. Of the 57 OAR-negative patients (those considered not to have ankle fractures based on the OAR), two had fractures that were detected on radiographic examination. Radiographs of all 183 injuries revealed only 63 fractures (34.4%); these included both significant fractures and avulsion fractures as shown in Table 1. The 63 fractures included 42 (66.7%) fractures of the lateral malleolus, 14 (22.2%) fractures of the medial malleolus, and seven (11.1%) fractures of both malleoli.

**Table 1:** Comparison of the Ottawa ankle rules and ankle radiographs for fracture detection in 183 patients with acute ankle injury.

Result of Ottawa ankle rules	Result of radiographs		total
	Fractures (+)	No fracture (−)	
Need radiography (+)	lateral malleolus 40 medial malleolus 14 bimalleolar 7	65	126
No radiography (−)	lateral malleolus 1 medial malleolus 1	55	57
total	63	120	183

In two cases, the OAR failed to predict small avulsion fractures; one of these was a small avulsion from the anterior tip of the medial malleolus and one was from the lateral malleolus. Significant fractures were defined as an avulsion fragment of greater than 3 mm [14]; none of these were missed when using the OAR for assessment. The sensitivity, specificity, and positive and negative predictive values of applying the OAR for predicting fractures of the malleolus were 96.8%, 45.8%, 48.4% and 96.5%, respectively. If the OAR had been used in our hospital setting to determine whether radiographs should be ordered, the number of radiographic examinations for acute ankle injuries would have been decreased by 31.1% (57/183). Two fractures (3.2%) would have been missed if the decision to perform radiographs was based solely on the OAR results.

The OAR are intended to be used as a clinical decision-making tool. If the results of examination using the OAR indicate that fracture is unlikely, the patients would not undergo radiographic examination to exclude fractures. In this study, 57 of the 183 patients enrolled would not have required subsequent radiographs after physical examination using the OAR. Based on the cost of standard ankle radiographs in our hospital, we calculated that this would have resulted in total savings of ¥4500 in the study. Additionally, both the patients with ankle injuries, and other patients requiring radiographs, would have spent less time in the hospital.

**Figure 2:**
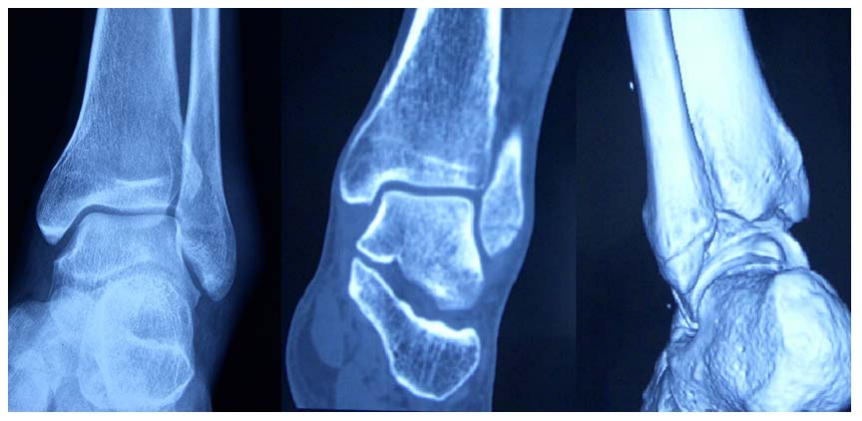
3D-CT images of a patient with high likelihood of fracture based on application of the OAR, but negative results of radiography. A fracture of the lateral malleolus can be clearly seen.

There were 23 patients with obvious soft-tissue swelling and severe tenderness over the bone that had no visible fracture on radiographs but were considered likely to have a fracture based on the OAR; these patients were advised to undergo a 3D-CT scan. Two of these patients refused further examination. Of the remaining 21 patients, five (23.8%) had occult fractures of the lateral malleolus that were detected by the CT scans (Figure 2).

## Discussion

Acute ankle injuries are very common and constitute 6–12% of ED visits as reported in the literature. Traditionally, radiographs are ordered for virtually all such patients, but only about 15% of these will have fractures. Thus, 85% of radiographic examinations for ankle injury are performed unnecessarily [15], [16]. Unnecessary radiographs result in increased radiation exposure, healthcare costs, and waiting time for the patient. An estimated $500,000,000 is spent annually on ankle radiography in Canada and the United States [17].

To reduce the number of unnecessary radiographs, Stiell *et al.* developed the OAR to help physicians decide which patients had a negligible risk of fracture and therefore no need for radiography [3], [4]. Since the rules were developed in 1992, they have been widely applied in many countries, and have undergone much study and development [6]–[14], [18]–[26]. Application of the OAR has been included in the curriculum for medical students at some medical schools.

Bachmann [27] summarized the available literature in an excellent systematic review. The application of the OAR results in a 30–40% reduction in the number of unnecessary radiographic examinations. Evidence supports the use of the OAR as an accurate clinical tool for excluding fractures of the ankle. The OAR has a sensitivity of almost 100%, and a modest specificity. The sensitivity of the OAR in our study was 96.8%. The specificity, positive predictive value, and negative predictive value for fractures of the ankle were 45.8%, 48.4% and 96.5%, respectively, and the reduction in unnecessary radiographic examinations that would have resulted from application of the OAR was 31.1%. These results are similar to those reported in previous studies [6]–[12], [27].

The sensitivity and specificity of the OAR are affected by both doctor and patient factors. Stiell *et al.* [3]–[5] indicated that the rules were easily learned, and general practitioners can master them after approximately 1 h of training. However, assessment of bone tenderness is subjective and depends upon factors such as the subtlety of the palpation technique and the pain tolerance of the patient. Some fractures can only be identified by palpation of the very tip of either malleolus. In our study, when using the OAR we missed two cases of avulsion fracture (one of the lateral and one of the medial malleolus). No significant fractures were missed. The two avulsion fractures were missed because the attending physicians did not palpate the tip of the malleolus because of obviously swollen soft-tissue. We therefore conclude that the tips of the malleoli must be palpated. Furthermore, physicians must carefully examine the entire distal 6 cm of the fibula because some spiral fractures exit proximally and might be missed if only the lateral malleolus is palpated. Regarding specificity, it is very important to distinguish between bone and soft-tissue pain. In our experience, if palpation-induced pain decreases during palpation of the same location for more than 3 s, then it may be soft-tissue pain; otherwise, it is bone tenderness. Sometimes it is necessary to repeat portions of the examination to obtain accurate results. Patient factors, including cultural norms, social status, economic and mental factors, and pain tolerance, can result in higher false positive or false negative rates.

In our study, the incidence of patients with acute ankle injury that had a fracture evident on radiographic examination was 34.4%; this is higher than the approximately 15% reported in the literature [1], [2]. This difference may be due to the following factors: (1) in China, many patients with ankle injuries would use traditional Chinese medicine rather than visit the ED of a hospital; and (2) many patients are worried about high medical costs and avoid seeking unnecessary treatment. Thus, in our population, many patients with slight or mild pain and soft-tissue swelling may be unwilling to go to the ED and were therefore not included in our study.

The OAR has proven to be a highly sensitive and modestly specific test for fractures associated with ankle injuries. Widespread application of the OAR by the medical community can decrease unnecessary ankle radiography and waiting time for patients. Implementation of the rules would result in significant savings in healthcare costs and medical resources without compromising quality of care. We support the introduction of the OAR into practice from both a clinical and a health policy point of view. In our opinion, the OAR should be introduced more widely, especially in developing countries and regions where medical resources are relatively scarce.

It is well known that some occult fractures are not detectable on radiographs. Thus, physical examination is very important for the detection of this type of fracture. Patients with sharp bone tenderness and severe soft-tissue swelling, but negative radiographic results, are considered to have a high fracture likelihood based on the OAR. We suggest that these ankles should be immobilized in a cast to prevent potential fracture displacement. In our study, 23.8% (5 of 21) of the patients who underwent 3D-CT were diagnosed with occult fractures of the lateral malleolus. The utility of the OAR for the detection of occult fractures of the malleolus that are not visible on radiographs may be their most important clinical application.

## Limitations

There are some limitations to this study. There were a limited number of patients in the study, and all of the patients were recruited from the ED of only one hospital. Thus our data may not be representative of the status of all ankle injuries in Shanghai. Also, not all of the patients with ankle injuries during the study period were recruited. The sample size for the study was determined based on the previously reported literature, and may not have been sufficient for our study population. Additionally, the patients who were advised and agreed to undergo a 3D-CT scan were not randomly selected. It is possible that there was selection bias, and some occult fractures may have been missed in patients that did not undergo 3D-CT. Finally, although all of the emergency orthopedists in our ED were trained in the application of the OAR, there are unavoidable individual differences in palpation technique and assessment of bone tenderness, which may have impacted the results of our study.
